# Interaction between the nervous and skeletal systems

**DOI:** 10.3389/fcell.2022.976736

**Published:** 2022-08-30

**Authors:** Jiajia Xu, Zhongmin Zhang, Junjie Zhao, Carolyn A. Meyers, Seungyong Lee, Qizhi Qin, Aaron W. James

**Affiliations:** ^1^ Department of Pathology, Johns Hopkins University, Baltimore, MD, United States; ^2^ Division of Spine Surgery, Department of Orthopaedics, Nanfang Hospital, Southern Medical University, Guangzhou, China; ^3^ Academy of Orthopedics, Guangdong Provincial Key Laboratory of Bone and Joint Degeneration Diseases, The Third Affiliated Hospital of Southern Medical University, Guangzhou, China; ^4^ Department of Physical Education, Incheon National University, Incheon, South Korea

**Keywords:** nervous system, skeletal system, crosstalk, bone repair and regeneration, nerve innervation

## Abstract

The skeleton is one of the largest organ systems in the body and is richly innervated by the network of nerves. Peripheral nerves in the skeleton include sensory and sympathetic nerves. Crosstalk between bones and nerves is a hot topic of current research, yet it is not well understood. In this review, we will explore the role of nerves in bone repair and remodeling, as well as summarize the molecular mechanisms by which neurotransmitters regulate osteogenic differentiation. Furthermore, we discuss the skeleton’s role as an endocrine organ that regulates the innervation and function of nerves by secreting bone-derived factors. An understanding of the interactions between nerves and bone can help to prevent and treat bone diseases caused by abnormal innervation or nerve function, develop new strategies for clinical bone regeneration, and improve patient outcomes.

## Introduction

The nerves are spatially connected to the skeleton. The skeleton is extensively innervated by sensory and sympathetic nerves (Togari et al., 2012). However, our understanding of the interrelationship between nerve and bone is still limited, which also hinders the treatment of neuro-skeletal disorders. Neuroskeletal regulation has received more attention in recent years, and some pioneering theories and ideas have been proposed. Skeletal function and regeneration were affected by neurological defects or mental disorders (Kelly et al., 2020; Yang et al., 2020). Furthermore, Ducy *et al.* found that bone remodeling was regulated by the central nervous system and its derived leptin (Ducy et al., 2000; Takeda et al., 2002), suggesting that nerves have the ability to modulate bone. Since then, researchers have explored the mechanisms by which neurons in the central and peripheral nervous systems communicate with cells in the skeletal microenvironment and have identified the contributions of sensory and sympathetic nerves to bone development, bone homeostasis, and bone remodeling (Grassel, 2014; Chen et al., 2019; Tomlinson et al., 2020; Wang et al., 2020). Moreover, the skeleton makes up approximately 15% of the total body weight and is one of the largest organ systems in the body, supporting the movement and protecting vital organs (Han et al., 2018; Su et al., 2019; Rath and Durairaj, 2022). However, in the last 20 years, it has gradually been discovered that the skeleton also acted as a secretory organ, regulating other tissue functions by secreting different bone-derived factors (Li et al., 2019; Qian et al., 2021). The crosstalk between bone and nerves is a hot topic of research currently. As part of this review, we will examine the role of nerves in bone formation and repair, analyze the regulation of the nervous system by the skeleton, and summarize the mechanisms of mutual regulation between nerves and bone.

### Nerve distribution within the bone

**FIGURE 1:**
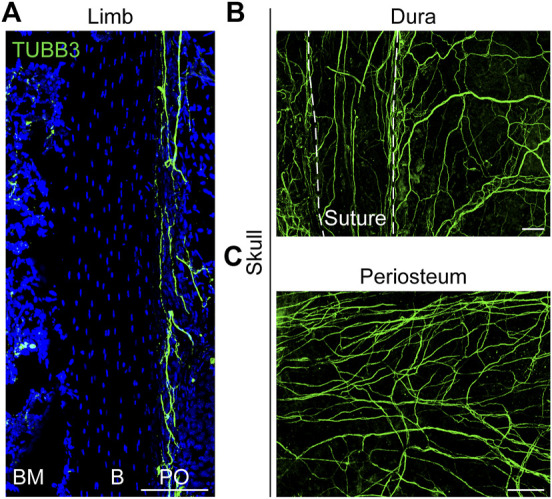
Distribution of nerves in limb and skull. **(A)** Pan-neuronal tubulin beta 3 Class III (TUBB3) whole-mount immunohistochemical staining of the mouse ulnar periosteum. **(B, C)** Whole-mount images of the mouse calvaria, showing the TUBB3^+^ nerve fibers in the **(B)** dura and **(C)** periosteum. White dashed lines indicate margins of the interfrontal suture, imaged from the endocranial aspect. B, bone; BM, bone marrow; PO, periosteum. White scale bar, 100 μm.

Connecting the central nervous system (CNS) to the skeleton is the peripheral nervous system (PNS), which acts as a transmitter of information. In the PNS, neurons are found in sensory and autonomic ganglia, collections of nerve cell bodies with axons that extend to target tissues (Brazill et al., 2019). Long bones and the calvaria are innervated by nerves that originate at different locations. Sensory neurons in the limbs originate in the dorsal root ganglion (DRG) next to the spinal cord and have a pseudo-unipolar morphology (Sorkin et al., 2018). Its axons bifurcate into peripheral projections that innervate the target tissue (Figure 1A) and can sense pain, pressure, heat and other stimuli and transmit them to central processing (Brazill et al., 2019). Nociceptors are the receptors that are sensitive to injurious stimuli (Dubin and Patapoutian, 2010). They can be further subdivided into peptidergic and non-peptidergic primary afferent neurons (Kestell et al., 2015). Nerve growth factor (NGF) is necessary for the survival and recruitment of peptidergic fibers, whereas fibers lacking peptides are dependent on glial-derived neurotrophic factor (GDNF) (Averill et al., 1995). Peptide-rich fibers expressing substance P (SP) and calcitonin gene-related peptide (CGRP) are prevalent in the skeleton of vertebrate species (Hill and Elde, 1991; Brazill et al., 2019). In addition, the collateral axon branches of these neurons can generate efferent signals that target their peripheral target tissues (Brazill et al., 2019). This local release of neuropeptides indicates the trophic nature of sensory nerves and their ability to modulate skeletal activity.

The autonomic nervous system (ANS) is a part of the peripheral efferent nervous system that innervates and regulates the activity of all organs of the body, including sympathetic and parasympathetic nerves (McCorry, 2007). The autonomic nerves in the limbs are mainly sympathetic (Brazill et al., 2019). Through preganglionic neurons in the spinal cord, autonomic nerves synapse with postganglionic neurons in the paravertebral ganglia of the sympathetic chain (Cherruau et al., 2003; Elefteriou, 2018). Sympathetic postganglionic neurons are noradrenergic, and they can project to a wide range of tissues in the body (Elefteriou, 2018). Sympathetic adrenergic neurons, which are abundant in bone, express tyrosine hydroxylase (TH) and promote the synthesis of norepinephrine (NE) (Hill and Elde, 1991; Mach et al., 2002). In addition, cholinergic neurons containing acetylcholine (ACh) and the neuropeptide vasoactive intestinal peptide (VIP) innervate bone (Hohmann et al., 1986; Cherruau et al., 2003).

Reports on nerve innervation in the skull are very limited. The ganglia that innervate the craniofacial skeleton are located in the cranial nerve ganglia (Brazill et al., 2019). Calvarial bone is surrounded by both intracranial (dura) and extracranial (periosteal) sheaths (Figures 1B,C) that contain sensory, sympathetic, and parasympathetic nerve endings (Kosaras et al., 2009). In the mature calvaria, the sutures are thought to serve as the major passageway of innervation between the dura and periosteum. From the dura, the fibers appear to penetrate the suture and emerge into the periosteum (Kosaras et al., 2009). Moreover, our results showed that the sutures were dominated by sensory nerves with a small portion of sympathetic nerves (Meyers et al., 2020). The regulatory mechanisms of neurodevelopment are complex. The influence of nerve formation and distribution on cranial repair remains to be elucidated.

In summary, the skeleton is covered by neural networks. Their location in space determines that they necessarily modulate each other. However, we know very little about the interaction between the skeleton and the nervous system, which has been a hot topic of research in recent years.

### Neural regulation of bone remodeling and repair

Bone is innervated by sensory nerve fibers expressing tropomyosin receptor kinase A (TrkA), which are required for the proliferation of osteochondral progenitors during skeletal development in mammals (Castaneda-Corral et al., 2011; Mantyh, 2014). Besides pain, little is known about the role of sensory innervation in bone regeneration. NGF is the first discovered neurotrophic factor that is primarily involved in the regulation of the growth, survival, as well as regeneration of neurons (Ebendal, 1992). In addition to directly activating TrkA^+^ sensory neurons to transmit injury signals, NGF determines the amount and type of innervation required for repair and regulates the growth of TrkA^+^ neurons into the cartilage membrane of long bones during embryogenesis, which is required for progenitor cell differentiation and bone mineralization (Reichardt, 2006; Tomlinson et al., 2016). Primary and secondary ossification centers exhibited delayed vascular invasion when TrkA^+^ signaling and innervation were inhibited, which further decreased the number of Osterix^+^ osteoprogenitor cells and reduced the length and volume of femur (Tomlinson et al., 2016). The regeneration of entire appendages by starfish and certain amphibians depends on nerves. We also found that mammalian fracture repair required TrkA signaling from sensory nerves (Li et al., 2019). NGF was significantly upregulated in the early stage of repair after fracture. The bone repair process involves the regulation of inflammation by macrophages and the formation of bone by mesenchymal stem cells (MSCs). In our study, the main sources of NGF production were periosteal MSCs and osteocalcin (OCN)^+^ bone-lining osteoblasts, followed by macrophages. TrkA^+^ nerve sprouting was positively correlated with NGF expression. The level of NGF and the number of newly generated nerve fibers peaked on day 3 and then dropped significantly. Moreover, the nerves in the callus were predominantly sensory and peptidergic fibers, with a small proportion of TH^+^ sympathetic nerve fibers. Inhibition of NGF-TrkA signaling impaired sensory nerve re-growth and vascular remodeling, leading to failure of bone healing. The research suggests that NGF regulates nerve ingrowth at an early stage, which facilitates vascular and bone regeneration. Thus, we reveal the sequence of cellular events that occur during re-innervation of fracture-healed tissue and demonstrate a critical role of TrkA sensory nerves in bone healing.

**FIGURE 2:**
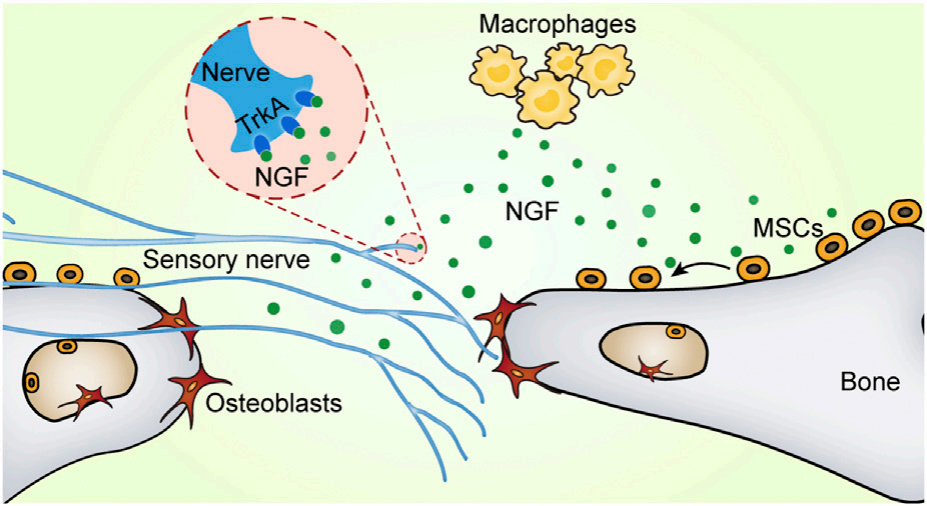
NGF promotes neural regeneration and MSC migration. There are two receptors for nerve growth factor (NGF): tropomyosin receptor kinase A (TrkA) and p75 (also known as CD271). Upon bone injury, NGF produced by macrophages activates the TrkA pathway to promote nerve fiber re-innervation. Furthermore, NGF recruits mesenchymal stem cells *via* the p75 signaling pathway to migrate toward bone defects and enhance bone formation.

The flat bones of the skull are densely innervated, which is essential for coordinated bone development and repair (Warren et al., 2003; Doro et al., 2017). Our group next characterized the innervation of the mouse skull and explored their role in cranial bone defect repair (Meyers et al., 2020). Nerve fibers were observed in the uninjured skull. During the early stages of calvarial defect repair, macrophages were activated and expanded rapidly, releasing significant amounts of NGF. Locally increased NGF promoted nerve regeneration and axonal ingrowth. Furthermore, immunohistochemical staining identified the sprouting nerve as primarily CGRP^+^ peptidergic nociceptors. The nerve regeneration process further promoted vascular remodeling and bone regeneration at the defect site. Like the long bone, TrkA is universally expressed on fibers that innervate the calvarium from the trigeminal ganglia. A mutation in TrkA protein inhibited NGF signaling and subsequently impaired the re-innervation of tubulin beta 3 Class III (TUBB3)^+^ nerve fibers. These findings suggest that nerve regeneration in the skull is also controlled by the NGF-TrkA signaling pathway. Blocking the NGF-TrkA signaling pathway led to reduced vascularity and decreased osteogenic capacity, which significantly delayed bone defect healing (Meyers et al., 2020). Interestingly, we found that NGF also regulated the migration of MSCs. Locally released NGF recruited the aggregation of MSCs to the defect site *via* the NGF-p75 signaling pathway, thereby coupling the neuro-skeletal interaction (Figure 2) (Xu et al., 2022). Despite these findings, we have yet to address the downstream molecular mechanisms of neuroskeletal intercommunication. It is particularly important to investigate how nociceptive fibers affect bone repair through secondary messengers.

Along with NGF, semaphorin 3A (Sema3A) is also a well-known axon guidance molecule (Tamagnone and Comoglio, 2004; Kruger et al., 2005; Fukuda et al., 2013). Neuron-derived Sema3A is essential for the normal development of peripheral neurons *in vivo* and contributes to nerve growth and regeneration (Zhang et al., 2018). Fukuda *et al.* demonstrated that Sema3A produced in neurons regulated bone remodeling by modulating sensory nerve innervation to the bone (Fukuda et al., 2013). Specific deletion of Sema3A in neurons resulted in lower bone mass, whereas mice with a Sema3A deficiency specific to osteoblasts had normal bone mass. Further studies revealed a significant reduction in the number of sensory innervations of trabecular bone in neuron-specific Sema3A-deficient mice, leading to skeletal abnormalities. In contrast, sympathetic nerve fibers, which inhibit the increase in bone mass, were not significantly affected. In combination with our transcriptome sequencing data (Xu et al., 2022), we found high expression of Sema3A locally in bone injury sites. Based on its role in bone remodeling, we hypothesize that Sema3A also plays an important regulatory role in bone repair by stimulating sensory nerve innervation and thus promoting bone regeneration. However, the relative roles of Sema3A and NGF in bone regeneration need to be further explored.

As the skeleton develops and fractures heal, nerve fibers are closely associated with blood vessels (Tomlinson et al., 2016; Li et al., 2019). Our study showed that nerve regeneration occurred before revascularization and contributed to vascular regrowth (Meyers et al., 2020). Angiogenesis is promoted by sensory nerve-released SP and CGRP *via* neurokinin-1 (NK1) and CGRPR receptors on endothelial cells (Marrella et al., 2018). We previously found that vascular extracellular vesicles (EVs) promoted MSC proliferation, migration, and differentiation and improved bone regenerative capacity (Xu et al., 2019). Thus, nerves can indirectly regulate bone repair through the vasculature in addition to their paracrine role in regulating bone regeneration.

**FIGURE 3:**
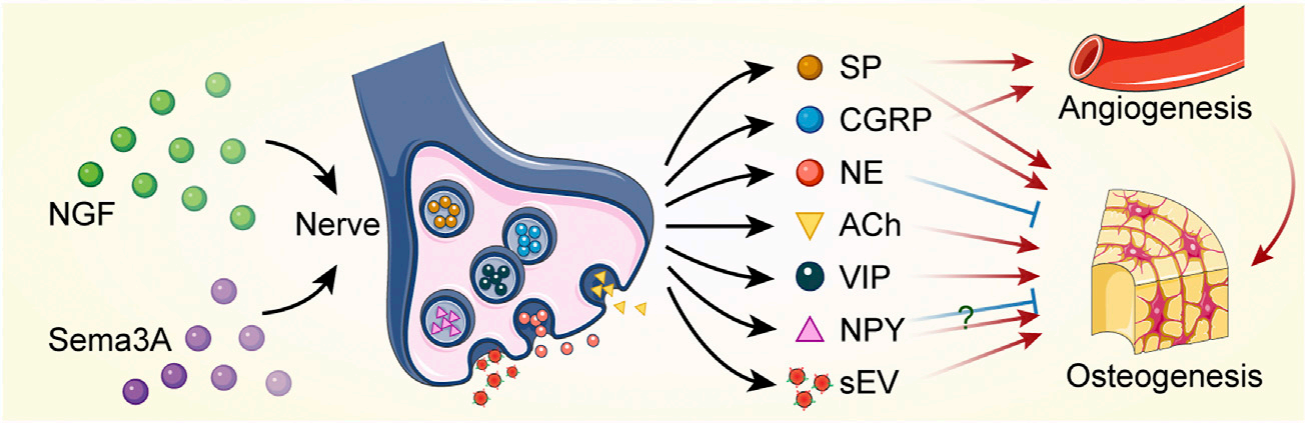
Bone regeneration is regulated by nerves through paracrine action. Nerve growth factor (NGF) and semaphorin 3A (Sema3A) promote nerve regeneration. Neurotransmitters and small extracellular vesicles (sEVs) are released by neural cells to regulate angiogenesis and osteogenesis. Red sharp arrows (→) indicate stimulation, while blue blunt arrows (┴) indicate inhibition. The role of neuropeptide Y (NPY) in regulating bone formation remains controversial. SP, substance P; CGRP, calcitonin gene-related peptide; NE, norepinephrine; ACh, acetylcholine; VIP, vasoactive intestinal peptide.

Clear nerve-derived factors mediating bone formation, including neurotransmitters and extracellular vesicles (Figure 3), have been elucidated in several *in vitro* studies and regenerative contexts. Bone lineage cells express receptors for neurotransmitters or neuropeptides that can be modulated by neurogenic substances. CGRP and SP, which are derived from sensory nerves, stimulate osteogenic differentiation and bone formation. CGRP is a critical and highly expressed sensory signal. It produces in both peripheral and central neurons and can function in the transmission of nociception (Rosenfeld et al., 1983; Crenshaw et al., 1987). CGRP promoted the proliferation of osteoprogenitor cells, reduced their apoptosis and enhanced the expression of osteogenic genes and the activity of osteoblasts *via* inhibiting NF-κB activation (Mrak et al., 2010; Wang et al., 2010). In addition, CGRP promoted the recruitment and cell proliferation of MSCs and enhanced osteogenic differentiation by activating mitogen-activated protein kinase (MAPK) signaling pathway (Yang et al., 2017; Wang et al., 2020) and upregulating the expression of alkaline phosphatase (ALP) and runt-related transcription factor 2 (Runx2) (Fang et al., 2013; Xu and Jiang, 2014). Following bone injury or fracture, CGRP-containing fibers may trigger perivascular bone regeneration. Local injection of CGRP was effective in accelerating bone formation (Jia et al., 2019). CGRP expression in the peripheral femoral cortex and dorsal root ganglion (DRG) was significantly increased following *in vivo* implantation of biodegradable magnesium, which promoted new bone formation (Zhang et al., 2016).

SP belongs to the tachykinin family and is a highly conserved 11-amino acid neuropeptide involved in pain perception (Hong et al., 2009; Steinhoff et al., 2014). SP is expressed in the peripheral nerves and is often released together with CGRP (Brazill et al., 2019). Similar to CGRP, SP has been shown to increase the proliferation and osteogenic differentiation of MSCs (Nagao et al., 2014). SP bound to and activated the NK-1 receptor (NK-1R), which was expressed by bone lineage cells (Goto et al., 1998; Goto et al., 2007; Wang et al., 2020). SP directly regulated bone metabolism through NK-1R (Goto et al., 1998). SP stimulated the proliferation of BMSCs in a concentration-dependent manner. Wang *et al.* found that low concentrations of SP promoted ALP activity and Runx2 expression, and high concentrations of SP contributed to matrix mineralization (Wang et al., 2009). Furthermore, SP enhanced MAPK, Wnt and bone morphogenetic protein 2 (Bmp2) signaling pathways as well as upregulated vascular endothelial growth factor (VEGF) expression, thereby promoting angiogenesis and osteogenic differentiation (Hong et al., 2009; Fu et al., 2014; Wang et al., 2020). SP also increased the mobilization of MSCs to the osteogenic region, accelerating bone remodeling and formation (Imai et al., 1997; Hong et al., 2009; Zhang et al., 2014).

Neuronal cells can also regulate bone regeneration remotely *via* extracellular vesicles. According to clinical studies, patients with craniocerebral injury and fractures have a faster callus formation and a shorter fracture healing time than patients with fractures alone. Xia *et al.* demonstrated that damaged neurons released small extracellular vesicles (sEVs) rich in pro-osteogenic microRNAs (miRNAs) after traumatic brain injury (TBI) (Xia et al., 2021). Fibronectin 1 on the membrane surface of sEVs might be a key protein for brain-derived sEVs to target bone tissue. Moreover, miR-328a-3p and miR-150-5p enriched in sEVs accelerated bone healing by regulating the transcription of FOXO4 and CBL proteins, respectively.

Sympathetic signaling reduces bone mass. Sympathetic tone activation upregulated intracellular OPN expression and induced bone loss. Mechanistically, intracellular OPN reduced the cAMP-response element binding (CREB) phosphorylation and osteogenic differentiation *via* the inhibition of β2-adrenergic receptor (Nagao et al., 2011). NE is the main neurotransmitter of the sympathetic nervous system and is synthesized from the amino acid tyrosine by the action of TH (Elefteriou, 2018). Adrenergic nerves release NE that inhibits bone formation. Its receptors include *a*- and *ß*-adrenergic receptors (ARs), such as β1, β2, α1B, α2A, α2B, and α2C (Grassel, 2014; Tank and Lee Wong, 2015; Brazill et al., 2019; Wan et al., 2021). NE participates in the regulation of bone remodeling mainly via its β2 receptor. Sympathetic signaling *via* β2-adrenergic receptors (β2-AR) present on osteoblasts controlled bone formation. Disruption of the noradrenergic signaling axis or knock of β2-AR increased bone mass (Elefteriou et al., 2005). Mechanistically, sympathetic tone signals in osteoblasts suppressed CREB phosphorylation *via* β2-AR, thereby reducing osteoblast proliferation (Takeda et al., 2002; Kajimura et al., 2011). Du *et al.* found that NE inhibited stem cell migration, downregulated Runx2 and OCN expression, and prevented the formation of mineralized bone nodules *via* β3-adrenergic receptor (Du et al., 2014).

Cholinergic nerve-derived ACh and VIP, as well as adrenergic nerve-produced neuropeptide Y (NPY) also play important roles in bone formation. ACh functions by binding to both muscarinic (mAChRs) and nicotinic acetylcholine receptors (nAChRs) (Jones et al., 2012). These receptors are expressed on bone lineage cells, suggesting that ACh may affect bone formation (Wan et al., 2021). Ma *et al.* demonstrated the beneficial effect of ACh signaling on bone mass increase, while it also promoted the maintenance of peak bone mass in adult mice (Ma and Elefteriou, 2020). Acetylcholinesterase (AChE) is a classical cholinergic hydrolase that mainly degrades acetylcholine. AChE decelerated bone growth by terminating the action of acetylcholine to balance acetylcholine concentrations at low levels, thereby promoting chondrocyte apoptosis and impeding bone mineralization. Conversely, reducing AChE increased ACh activity and promoted bone formation (Luo et al., 2020). VIP is normally released from cholinergic neurons together with acetylcholine. There are two known receptors for the VIP termed VPAC1 and VPAC2. VIP promoted osteogenic differentiation of MSCs by activating the Wnt/β-catenin signaling pathway. VIP also increased the expression of VEGF in MSCs to stimulate angiogenesis. Additionally, VIP significantly boosted cranial defect repair, bone formation, and angiogenesis *in vivo* (Shi et al., 2020). PKA activation by VIP resulted in the accumulation of cAMP and affected multiple signaling pathways that regulate osteoblast differentiation (Juhasz et al., 2016). VIP also upregulated ERK 1/2 activation in osteoblasts (Juhasz et al., 2015). NPY is usually accompanied by the release of NE in sympathetic nerves (Wan et al., 2021). NPY acts through five G protein-coupled receptor isoforms, Y1R, Y2R, Y4R, Y5R and Y6R, of which Y1R and Y2R are usually involved in bone mass regulation (Chen and Zhang, 2022). Osteoblast-specific Y1 receptor knockout mice exhibited high bone mass, suggesting that the NPY-Y1R signaling axis regulates bone homeostasis (Yahara et al., 2017). Reduced Runx2 levels and mineralized nodules after NPY-treated MSC osteogenesis confirmed that NPY impaired osteogenic differentiation (Zhang et al., 2021). Blockade of NPY enhanced bone mass (Baldock et al., 2009; Lee et al., 2010; Xie et al., 2020). However, some studies have also reported a positive role for NPY in bone formation and bone repair (Chen and Zhang, 2022). Low doses of NPY promoted osteogenesis and mineralization in MSCs, whereas high concentrations of NPY had the opposite effect (Liu et al., 2016). NPY overexpression significantly upregulated osterix and Runx2 levels and enhanced osteogenic capacity (Zhang et al., 2020). The specific regulatory role of NPY on bone formation needs to be further clarified.

Nevertheless, our understanding of the exact key molecules released from nerve fibers within a bone injury site that regulate bone regeneration *in vivo* remains unclear and is the subject of continued investigation.

## Skeletal regulation of neural function

Bone has long been thought to serve only as a supportive and protective organ (Zhu et al., 2020). In the last 20 years, it has slowly become apparent that bone can also act as an endocrine organ by secreting various bone-derived factors that have regulatory effects on the central nervous system, immune system, energy metabolism, etc (Guntur and Rosen, 2012; Moser and van der Eerden, 2018; Du et al., 2021). OCN is a marker gene for osteogenic differentiation and is synthesized solely in osteoblasts (Klingelhoffer et al., 2016; Moser and van der Eerden, 2018). It plays an important role in regulating bone calcium metabolism. OCN is considered as a multifunctional bone-derived hormone that regulates a variety of physiological activities and developmental processes (Karsenty and Olson, 2016; Obri et al., 2018; Qian et al., 2021). OCN regulates the neuronal function and is required for both brain development and brain function in mice (Obri et al., 2018). Khrimian *et al.* found that the target receptor for OCN in the brain was the G-protein coupled receptor Gpr158, which was expressed in neurons in the CA3 region of the hippocampus (Khrimian et al., 2017). Despite this, Gpr158 was not expressed in all OCN-active brain regions, suggesting that other receptors respond to osteocalcin signaling in the mouse brain. Recently, Qian *et al.* demonstrated that OCN regulated the transition of oligodendrocyte precursor cells (OPCs) into mature oligodendrocytes (OLs) and was essential for OL myelination and remyelination after demyelination injury (Qian et al., 2021). OCN deficiency led to upregulated myelin-related gene expression and excessive myelin formation in the central nervous system. G Protein-Coupled Receptor 37 (GPR37) was abundantly expressed in mature OLs and was a novel specific receptor for OCN. OCN suppressed myelin gene regulatory factor (Myrf) through GPR37, which was required for OCN to regulate OL differentiation and myelination. This study reveals reciprocal crosstalk between bone secreted-OCN and glial cell function, providing evidence for an important role of OCN in regulating OL myelin formation. Furthermore, osteoblast-derived OCN could cross the blood-brain barrier and bind to neurons in the brainstem, midbrain, and hippocampus, enhancing the production of monoamine neurotransmitters, preventing anxiety and depression, and facilitating learning and memory. In addition to these, before the embryos synthesized OCN, the mother delivered this hormone to the fetus *via* the placenta to prevent neuronal apoptosis (Oury et al., 2013).

**FIGURE 4:**
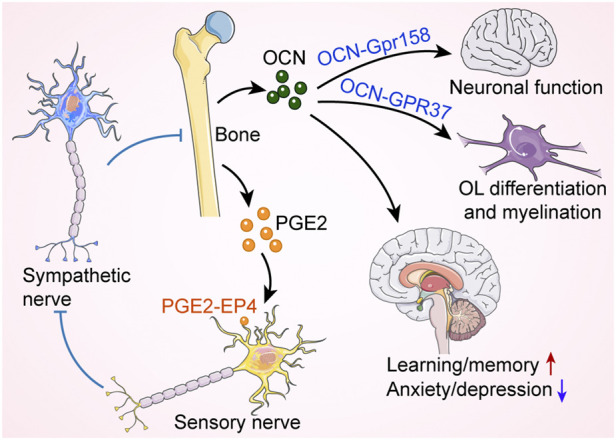
Bone regulates neural function and activity. Osteocalcin (OCN) released from skeleton controls oligodendrocyte (OL) differentiation and neural function through G-protein coupled receptors, affecting cognition and mood. Moreover, bone produces prostaglandin E2 (PGE2), which binds to EP4 receptors on sensory nerves to inhibit sympathetic nerve activity, thereby promoting bone formation. Red arrow indicates an increase, while blue arrow indicates a decrease. Blue blunt arrows (┴) indicate inhibition.

Increased prostaglandin E2 (PGE2) levels in osteoporotic mice were accompanied by a significant reduction in CGRP^+^ sensory fibers (Chen et al., 2019). PGE2 receptor 4 (EP4) is the main receptor for PGE2 (Hawcroft et al., 2007; Konya et al., 2013), which is expressed in bone sensory nerves. Chen *et al.* clarified that PGE2 secretion by osteoblasts was elevated under conditions of reduced bone mass, and this signal, transmitted through EP4, regulated osteoblast-mediated bone formation (Chen et al., 2019). The specific mechanism was that PGE2 stimulated hypothalamic CREB signaling through EP4 in sensory nerves to inhibit sympathetic tone, which in turn regulated osteoblast differentiation and promoted bone regeneration (Figure 4).

During early embryonic development in mice, NGF produced by osteochondral progenitor cells promoted skeletal nerve innervation and function by activating TrkA signaling, which directed sensory nerve axons to the site of initial ossification (Tomlinson et al., 2016). In a long bone stress fracture model, we found that periosteal MSC- and osteoblast-derived NGF could regulate the reinnervation of nerves, representing the role of skeletal regulation of nerves after injury (Li et al., 2019). In contrast, MSC-derived NGF was not a key factor in regulating nerve regeneration during calvarial defect repair (Meyers et al., 2020), suggesting that the mechanisms regulating nerve ingrowth during regeneration differ depending on the physiological site.

## Conclusion

The peripheral nerves in the skeleton include sensory and sympathetic nerves. The mechanisms of skeletal-neural crosstalk are complex, and a rich variety of cells and factors are involved. There are still many unknown regulatory effects. In this review, we explore the role of nerves in the regulation of bone remodeling and repair and parse the effect of bone as an endocrine organ on nerves. In our research, we are more interested in the mechanisms of neural regulation of the skeleton. Innervation of bone occurs almost simultaneously with endochondral ossification during embryonic development (Bidegain et al., 1995; Sisask et al., 2013). The increase in nerve density in bone coincides with bone growth and remodeling, forming a dense network covering the bone (Tomlinson et al., 2020). Bone injury is accompanied by nerve damage, and nerve reinnervation is essential for bone repair. We found that MSCs, osteoblasts and macrophages all secreted NGF. NGF-expressing cells that play a key role in long bone and calvarial bone injuries are differential. The specific roles of skeletal and immune cells in injury repair and neural regeneration remain to be investigated. Moreover, the results from single-cell studies revealed that NGF was highly expressed in perivascular stem cells (especially pericytes) and that the NGF-TrkA signaling pathway promoted angiogenesis and vascular regeneration (Graiani et al., 2004; Vera et al., 2014; Ahluwalia et al., 2016). The fact that EVs of vascular origin could stimulate stem cell activity and regenerate skeletal tissues suggests the diversity of NGF sources and its multiple roles. In this regard, further research is required.

Signals from nerves can promote bone formation and prevent bone degeneration. Targeting pro-neural regenerative pathways or specific signals from peripheral nerves can accelerate bone healing. Dissecting the neuro-skeletal crosstalk could advance our understanding of bone metabolism and the pathogenesis of diseases such as osteoporosis, osteoarthritis, heterotopic ossification and bone tumors, as well as identify potential targets for the mitigation and prevention of these diseases. Related research could also contribute to the development of novel bone repair materials. Materials that target both nerve and bone regeneration could offer new hope and solutions to major clinical challenges, such as large bone defects.
